# Deacetylation of sialic acid by esterases potentiates pneumococcal neuraminidase activity for mucin utilization, colonization and virulence

**DOI:** 10.1371/journal.ppat.1006263

**Published:** 2017-03-03

**Authors:** Hasan F. Kahya, Peter W. Andrew, Hasan Yesilkaya

**Affiliations:** 1 Department of Infection, Immunity & Inflammation, University of Leicester, United Kingdom; 2 Department of Biology, College of Education, University of Mosul, Iraq; University of Birmingham, UNITED KINGDOM

## Abstract

Pneumococcal neuraminidase is a key enzyme for sequential deglycosylation of host glycans, and plays an important role in host survival, colonization, and pathogenesis of infections caused by *Streptococcus pneumoniae*. One of the factors that can affect the activity of neuraminidase is the amount and position of acetylation present in its substrate sialic acid. We hypothesised that pneumococcal esterases potentiate neuraminidase activity by removing acetylation from sialic acid, and that will have a major effect on pneumococcal survival on mucin, colonization, and virulence. These hypotheses were tested using isogenic mutants and recombinant esterases in microbiological, biochemical and *in vivo* assays. We found that pneumococcal esterase activity is encoded by at least four genes, SPD_0534 (EstA) was found to be responsible for the main esterase activity, and the pneumococcal esterases are specific for short acyl chains. Assay of esterase activity by using natural substrates showed that both the Axe and EstA esterases could use acetylated xylan and Bovine Sub-maxillary Mucin (BSM), a highly acetylated substrate, but only EstA was active against tributyrin (triglyceride). Incubation of BSM with either Axe or EstA led to the acetate release in a time and concentration dependent manner, and pre-treatment of BSM with either enzyme increased sialic acid release on subsequent exposure to neuraminidase A. qRT-PCR results showed that the expression level of *estA* and *axe* increased when exposed to BSM and in respiratory tissues. Mutation of *estA* alone or in combination with *nanA* (codes for neuraminidase A), or the replacement of its putative serine active site to alanine, reduced the pneumococcal ability to utilise BSM as a sole carbon source, sialic acid release, colonization, and virulence in a mouse model of pneumococcal pneumonia.

## Author summary

Neuraminidase activity is critical for pneumococcal colonization and virulence as it is required for efficient cleavage of host glycans for nutritional requirements, attachment, and translocation of the microbe through biological membranes. Modifications, such as *O*-acetylation, in terminal sialic acid can affect the potency of neuraminidase. In this study we investigated whether pneumococcal esterases could potentiate neuraminidase activity by de-acetylating sialic acid. We found that the pneumococcal esterase activity is coded by at least four genes, specific for short acyl chain esters, and the removal of acetylation by esterases potentiates pneumococcal neuraminidase activity for mucin utilisation, colonization and virulence. Hence, this study elucidates the complexity and importance of host de-glycosylation for pneumococcal colonization and virulence, and reveals a potential target for therapeutic intervention.

## Introduction

Glycosylation is the most common posttranslational modification for proteins and lipids [1, 2]. Given the abundance of the glycans, microbes have evolved mechanisms to take advantage of the carbohydrate-rich environment in the human body during colonization and, invasive infections [2]. *Streptococcus pneumoniae* is a good example in case, which has been used as a model organism for the study of microbial interaction with the glycosylated host molecules [3–5]. This microbe is a commensal of the nasopharyngeal microbiota but it also causes serious life-threatening infections with a high morbidity and mortality, such as meningitis, bacteraemia, and pneumonia [6, 7]. The pneumococcus encounters glycosylated host molecules both during colonization and infection of various tissues, and these molecules are very relevant for pneumococcal *in vivo* survival, attachment, and invasiveness [4, 8–10]. Therefore, a detailed understanding of pneumococcal interaction with host glycoconjugates can offer strategies to combat this pathogen.

The pneumococcus generates energy through fermentative metabolism of sugars. However, in the respiratory tract the concentration of free sugars is limited [11]. Therefore, the pneumococcus relies on glycosylated host molecules for its nutrient requirements [11]. The hydrolysis of sugars from the complex host glycans is achieved by a collection of glycosidases [12–15]. Not surprisingly, the pneumococcus has at least 10 exo-glycosidases, including galactosidase, heaxosaminidase, and neuraminidases [8]. By its glycosidases, *S*. *pneumoniae* has capability to reduce complex glycans with the exo-glycosidases neuraminidase, galactosidase, and hexosaminidase to remove the terminal sialic acid, galactose, and heaxosaminidase, respectively. Cleavage of sugars from host glycoconjugates does not only provide *in vivo* source of nutrients, but also it allows the pneumococcus to infiltrate into deeper tissue sites and translocate from one niche to another [8, 16].

Neuraminidase, also known as sialidase, cleaves terminal sialic acid from glycoconjugates, and is the most crucial sugar hydrolase since the cleavage of terminal sialic acid is required for sequential degradation of host glycoconjugates [3, 16]. Unless it is removed, the other sugar hydrolases cannot ‘see’ their substrate. In *S*. *pneumoniae* the total neuraminidase activity is encoded by three different genes, *nanA*, *nanB* and *nanC* [16–20]. *nanA* is present in all pneumococcal strains, while *nanB* and *nanC* are reported to be in 97% and 51% of isolates, respectively [21]. The major pneumococcal neuraminidase activity is NanA, which has cell surface localization, and cleaves α2-3- and α2-6-linked sialic acid [15, 20, 22].

Neuraminidase A (NanA) plays an important role in pneumococcal colonization and virulence as it decreases the density of mucin, which mediates the pneumococcal infiltration into deeper tissue sites [15], and exposes the host receptors for the pneumococcal attachment [23]. By using different animal models of pneumococcal infection, neuraminidase A has been linked to otitis media, meningitis, and upper and lower respiratory tract infection, and sepsis [15, 24, 25]. Therefore, the study of factors important for potency of NanA provides a better understanding of pneumococcal colonization and virulence.

The activity of neuraminidase can be hindered due to the modifications present in sialic acid [26]. The most frequent derivatives of sialic acid in human cells is N-acetyl-9-*O*-acetylneuraminic acid (Neu5,9Ac_2_), N-acetylneuraminic acid (Neu5Ac) and N-glycolylneuraminic acid (Neu5Gc) [27–29]. After synthesis, sialic acid is modified in the Golgi apparatus of eukaryotic cells [26, 30], and the modifications include *O*-acetylation, N-acetylation, methylation, hydroxylation and lactylation [26]. Among these *O*-acetylation is the most common modification, and one sialic acid molecule may carry up to four acetyl residues at 4-, 7-, 8- and 9-C [31]. *O-*acetyl groups can influence sialic acid metabolism by increasing sialic acid synthesis and critically by preventing sialic acid cleavage from glycoconjugates [32]. It has been reported that the activity of sialidases is increased by 50–80% when all *O*-acetyl group are removed from sialic acid [33, 34]. Given that up to 10% of human nasal mucin is acetylated [35], the pneumococcal ability to deal with acetylation must be important for the infections caused by the microbe. Removal of acetylation by esterases could be a main step of sialic acid removal by neuraminidase A. However, how the acetylation affects neuraminidase A activity, and more widely, what impact this modification may have in pneumococcal colonization and virulence are not known.

Esterases are hydrolytic enzymes that can liberate acetyl groups from partially acetylated substrates. Evidence regarding esterase activity against *O*-acetyl groups on terminal sialic acid has been studied with viruses [36]. But very little is known about how bacterial pathogens de-acetylate sialic acid, and how this may effect host-pathogen interaction. We hypothesised that esterases are important for pneumococcal growth on host glycoconjugates, and virulence through potentiation of neuraminidase activity. In this study, the pneumococcal esterases have been characterised by biochemical assays, and their role in augmentation of neuraminidase activity was tested using genetically modified strains *in vitro* and *in vivo*.

## Results

### At least four genes are responsible for pneumococcal esterase activity

*In silico* analysis showed that the sequenced *S*. *pneumoniae* strain D39 (serotype 2) has four putative esterases: acyl-ACP thioesterase (SPD_1239), tributyrin esterase (*estA*, SPD_0534), phosphoesterase (SPD_0932), and acetyl-xylan esterase (*axe*, SPD_150*6*) [37]. To determine the contribution of each of them to the total pneumococcal esterase activity, isogenic mutants were constructed and tested using chromogenic substrates. Analysis indicated that all isogenic mutants (42.5±1.6, 16.9±0.7, 42.8±1.2 and 33.8±1.2 mU for *ΔSPD1239*, *ΔestA*, *ΔSPD0932* and *Δaxe*, respectively, n = 6 for all strains) had significantly lower esterase activity than the wild type (50.0±1.6 mU, n = 6) when 4-Nitrophenyl acetate (pNPA) was used as a substrate (p<0.01). The largest reduction in activity was observed in *ΔestA*, indicating that EstA is the major pneumococcal esterase. Mutation of both *estA* and *axe* in *ΔestAaxe* (4.1±0.76 mU, n = 6) further reduced the activity (p<0.05).

When 4-Nitrophenyl butyrate (pNPB) was used as a substrate, which is a 4-carbon acyl ester, while the activity in *ΔSPD1239* (19.8±0.3 mU, n = 6) and *ΔSPD0932* (19.1±0.2 mU n = 6) was similar to the wild type (21.2 mU±1.1, n = 6) (*p*>0.05), *ΔestA* (7.3±0.4 mU, n = 6) and *Δaxe* (14.6±0.6 mU, n = 6) had significantly lower activity than the wild type (p<0.01 for both). These results show that esterase encoded by SPD_1239 and SPD_0932, are specific for short-chain acyl esters, while *estA* and *axe* encode esterases that are active against both 2- and 4-carbon acyl esters. In the genetically complemented strains estAComp (44.2±2.3 mU, n = 6) and axeComp (48.2±1.1 mU, n = 6), the esterase activity was similar to the wild type level when assayed with pNPA (p>0.05), indicating that the mutation of these genes did not cause polar effects.

### EstA and Axe are serine dependent enzymes

Homology comparison of EstA and Axe with known esterases indicated that they are serine dependent enzymes because both contained a typical catalytic triad of Ser-His-Asp, and have a characteristic consensus sequence of Gly-X-Ser-X-Gly (where X represents an arbitrary amino acid residue) around the active site serine (**S1 Fig**). Serine residue within a catalytic triad of Ser-His-Asp has been reported to play a key role for enzymatic activity of esterases [38]. The importance of this site has been studied neither in EstA nor in Axe. Hence, the putative EstA and Axe as well as their genetically modified versions with putative serine active site replacement were purified to test their substrate specificity in detail, to determine the kinetic properties, and to study the importance of serine within catalytic triad for the activity.

To verify whether EstA and Axe are serine dependent enzymes, we introduced mutations to replace the serine at positions 121 and 181 in EstA and Axe, respectively, with alanine. The results showed that the strains carrying these modifications in EstA and Axe, estAComp^S121A^ (14.8±0.5 mU, n = 6) and axeComp^S181A^ (28.4±0.65 mU, n = 6), had lower esterase activity than the wild type (50.02±1.56 mU, n = 6) (p<0.01). These results indicate that the serine is important for the catalytic activity of both EstA and Axe, consistent with other serine esterases [38].

### Evaluation of Axe and EstA with chromogenic substrates

**Fig 1:**
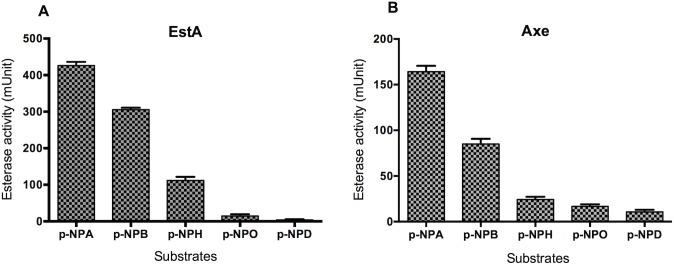
**Activity of recombinant EstA (A) and Axe (B) against different types of p-NP esters.** One mU of enzyme activity is expressed as micromole of p-nitrophenol released from the substrate per milligram of protein per minute. Each column represent the mean of data derived from at least four independent experiments in replicates. The vertical bars are for the standard error of mean.

To study, the substrate specificity in further detail, the activity of recombinant Axe and EstA was tested against a wider range of chromogenic substrates. The activity of both enzymes against different chromogenic substrates of varying acyl chain length (pNPA, pNPB, pNPH, pNPO and pNPD, with 2, 4, 6, 8, and 10 C, respectively) was determined at their optimal pH of 7.5. It was found that there was a reverse correlation between the activity level and carbon chain length (**Fig 1**). For example, the specific activity against pNPA was 426.1± 4.1 mU and 164.3±3.2 mU for EstA and Axe, respectively, and for pPND it was 7.2 ± 0.9 mU, and 9.5 ± 1.848 mU (n = 6 for all), for EstA and Axe, respectively. Using pNPA, the *Vmax* and *Km* values for EstA were calculated to be 364.1±13.9 mU and 4.7±0.6 mM, respectively, and for Axe the values were 220±7.94 mU and 4.4±0.58 mM, respectively.

### Evaluation of Axe and EstA against natural substrates

**Fig 2:**
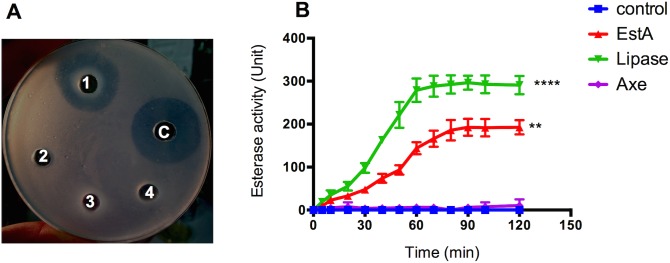
**Qualitative (A) and quantitative (B) esterase activity assay using tributyrin (triglyceride) as substrate.** (**A**) 1: EstA; 2: EstA^S121A^; 3: Axe; 4: Axe^S181A^; C: *S*. *aureus* lipase. 50 μg protein was added into each well. (**B**) The enzyme activity is expressed as micromoles of fatty acids released from the substrate per microgram of protein per minute. **p<0.01, ****: p<0.0001 compared to control (no enzyme). Data derived from 3 independent experiments.

Although both Axe and EstA could use chromogenic substrates, we determined whether EstA and Axe could use tributyrin (triglyceride) and acetylated xylan as substrates, respectively, as the genome annotation suggested. EstA and Axe were tested on tributyrin, a substrate for carboxylesterases [39], and acetylated xylan, a substrate for carbohydrate esterases that can utilise *O*-acetyl substituents within the substrate [40]. As shown qualitatively in **Fig 2A**, the recombinant EstA could use tributyrin as a substrate. A zone of clearance representing a positive reaction can be seen, and this was comparable to the zone of clearance (26.8 mm) obtained with 300 U of commercial lipase from *Staphylococcus aureus*, which can also use tributyrin as a substrate [39]. On the other hand, neither EstA^S121A^ nor Axe was active against tributyrin. This suggests that EstA belongs to carboxylesterase family and that serine S^121^ is important for EstA catalytic activity (**Fig 2A**). The specific activity of Axe and EstA against tributyrin was also measured spectrophotometrically. It was found that the specific activity of EstA was 198.3±1.7 U per mg activity when tributyrin was used as a substrate. As before, Axe did not display any activity towards tributyrin (**Fig 2B**).

**Fig 3:**
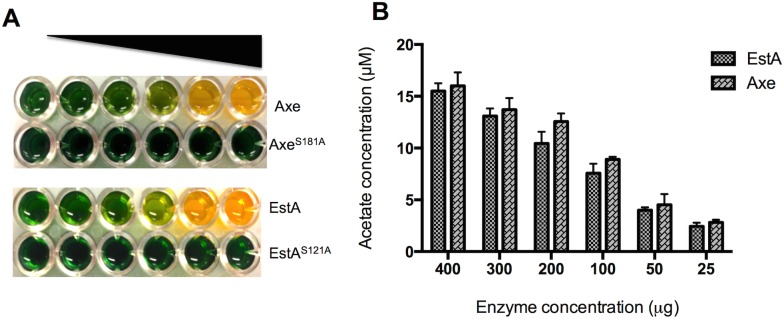
The catalytic activity of recombinant esterases on acetylated xylan as substrate. **A.** Axe and EstA activity on acetylated xylan. 25–400 μg esterase range was used for the assay. **B:** Concentration dependent esterase activity using acetylated xylan as the substrate. The assay was set up at pH 7.3 and different concentrations of enzyme used. The amount of released acetate in the reaction mixture was measured by using a standard curve generated with known concentrations of acetate.

The specific activity of EstA and Axe against acetylated xylan was also determined. The results are shown in **Fig 3A** and **3B**. It was found that when 400 μg of either enzyme was used, the amount of acetate release was 15.5±0.7- and 16±0.6 μM for EstA and Axe, respectively, indicating that both EstA and Axe are equally effective in utilization of acetylated xylan as substrate. Replacement of the serine 121 of EstA and 181 of Axe abolished the catalytic activity (**Fig 3**).

### EstA and Axe can use bovine submaxillary mucin (BSM) as a substrate

BSM contains up to 17% sialic acid, and 22.5% of this is reported to be acetylated [41]. Esterase activity on BSM was measured by quantifying the acetate released from the substrate. The results showed a positive correlation between acetate release and incubation time for both esterases (**S2 Fig**). The released acetate concentration at 120 min was significantly higher than that of at 30, 60 and 90 min (p<0.01). The replacement of S^121^ and S^181^ in EstA (13±1.5 μg/ml, n = 5) and Axe (12.8±2.4 μg/ml, n = 5), respectively, abolished acetate release.

### Axe and EstA potentiate NanA activity

**Fig 4:**
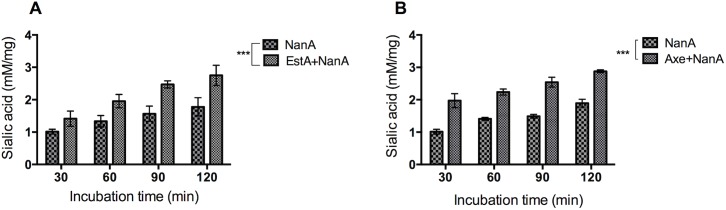
**Time dependent sialic acid release from BSM by neuraminidase with or without EstA (A) or Axe (B) treatment.** Each assay was done five times in triplicate. *** p<0.001 compared to NanA treatment alone.

Synergism between esterases and NanA was investigated. Firstly, BSM was incubated with 250 U of recombinant Axe or EstA for 90 min, and then incubated for an additional 90 min with 250 U recombinant NanA. The result showed higher sialic acid release by NanA when BSM had been pre-treated either with Axe (2.81±0.34 mM/mg, n = 5) or EstA (2.78±0.44 mM/mg, n = 5), than NanA treatment alone (1.78±0.33 mM/mg, n = 5) (p<0.01 for both). However, treatment of BSM either with EstA^S121A^ (1.83±0.75 mM/mg, n = 5) or Axe^S181A^ (1.71±0.65 mM/mg, n = 5) did not have any effect on sialic acid release, indicating the importance of catalytic activity rather than the absence of protein. Treatment with EstA (0.81±0.13 mM/mg, n = 5) or Axe (0.75±0.21 mM/mg, n = 5) alone did not result in detectable sialic acid release. Moreover, the increase in pre-treatment time with esterases enhances the release of sialic acid from BSM by NanA as shown in **Fig 4A** and **4B**. For example, while the amount of released sialic acid was 1.5±0.4 mM/mg and 1.5±0.1 mM/mg after 30 min pre-treatment, at 120 min the released sialic acid concentration increased to 3.0±0.3 mM/mg and 2.9±0.6 mM/mg for EstA and Axe, respectively (p<0.001).

### *ΔestA*, but not *Δaxe*, is attenuated in growth on BSM as sole carbon source

Having established the esterases’ role in potentiation of neuraminidase activity in cell free system, next, we determined whether esterases could potentiate NanA activity in intact cell. For this, the growth of *ΔestA*, *Δaxe* and *ΔnanA* mutants were compared to those of *ΔestAnanA* and *ΔaxenanA* mutants on BSM as the sole carbon source. In addition to growth, the released sialic acid in culture supernates was determined. This was possible because D39 is defective in utilisation of sialic acid [42], hence any released sialic acid remains in culture supernatant.

**Fig 5:**
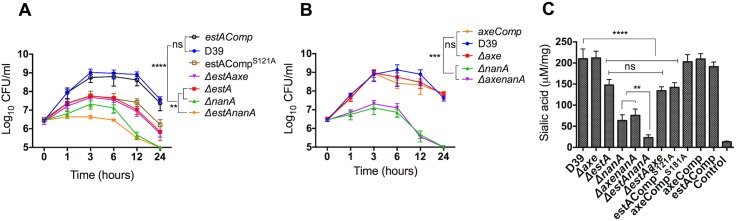
**Growth profiles of *ΔestA* (A), and *Δaxe* (B) in CDM supplemented with 0.071% (w/v) of BSM as carbon source, and sialic acid quantification in spent culture supernates of pneumococcal strains (C).** Each datum point represents the mean of five independent tests in replicates, and the vertical lines represent the standard error of mean. The pneumococcal strains were grown overnight on CDM supplemented with BSM as sole carbon source, and sialic acid levels in culture supernatant were measured. ‘ns.’ not significant; ** p<0.01, *** p<0.001, **** p<0.0001 compared to wild type or double mutant.

It was found that 0.071% (w/v) BSM was the optimal for pneumococcal growth. The growth profiles of pneumococcal strains on 0.071% BSM are shown in the **Fig 5A** and **5B**. After 6 h growth the colony counts for *ΔestA* (Log_10_ 7.4±0.27 CFU/ml, n = 5), estAComp^S121A^ (Log_10_ 7.7±0.55 CFU/ml, n = 5), and *ΔnanA* (Log_10_ 6.9±0.13 CFU/ml, n = 5) were significantly lower than that of wild type (Log_10_ 9.1±0.87 CFU/ml, n = 5) and estAComp (Log_10_ 8.85±0.34 CFU/ml, n = 5) (p<0.0001). As expected, there was no significant difference in growth profiles of D39 and estAComp (p>0.05). The major attenuation in growth, however, was seen in *ΔestAnanA* as the Log_10_ CFU/ml of double mutant decreased progressively during the time course of growth. Furthermore, the growth rate of double *ΔestAnanA* mutant was significantly lower than *ΔestA* and *ΔnanA* mutants (p<0.01). On the other hand, Δ*axe* grew as well as the wild type on BSM, and there was no difference in bacterial counts between *ΔaxenanA* and *ΔnanA* (p>0.05) (**Fig 5B**).

The sialic acid concentration in culture supernatants was consistent with the growth profiles of pneumococcal strains. The highest level of sialic acid was released by D39 (212±10.3 μM/mg, n = 5) and estAComp (195.4±5.3 μM/mg, n = 5) strains, and the lowest was with the *ΔestAnanA* (25.3±1.3 μM/mg, n = 5) compared to D39 (p<0.0001) (**Fig 5C**). Moreover, the released sialic acid concentration from *ΔestA* (143.5±6.3 μM/mg, n = 5), estAComp^S121A^ (138.7±4.5 μM/mg, n = 5) and *ΔnanA* (65.8±7.5 μM/mg, n = 5) culture supernates was significantly lower than the wild type, but higher than *ΔestAnanA* (*p*<0.01). On the other hand, the level of sialic acid from *Δaxe* (209.2±8.3 μM/mg, n = 5) culture supernate was as high as the wild type (p>0.05) (Fig 5). Moreover, there was no significant difference in sialic acid release between *ΔestAaxe* and *ΔestA* (p>0.05), ruling out compensation of esterase activity by Axe in the *ΔestA* cohort.

### EstA and Axe have an intracellular localization but esterase activity also can be detected in the culture supernates

To determine the subcellular localization of pneumococcal esterases, pneumococci were fractionated and hybridised with anti-EstA and anti-Axe polyclonal antibodies. To determine the efficiency of cell fractionation, lactate dehydrogenase (LDH) activity, known to have intracellular localization, was determined in different cell fractions and culture supernatant. It was found that the soluble intracellular fraction contained the highest LDH activity (357.5±21 mU/mg) compared to cell membrane (20.8±11.4 mU/mg) and cell wall fractions (9.4±3.6 mU/mg), indicating the quality of fractionation.

Both polyclonal antibodies reacted strongly with the recombinant EstA and Axe regardless of pneumococcal growth on BSM or glucose (**S3 Fig**). Each antibody reacted well with the soluble intracellular fraction, whereas there was no binding to the cell membrane and cell wall fractions. In addition, esterase activity was also detected in culture supernatant of *S*. *pneumoniae* (18.1± 1.3 mU, n = 5) (S3 Fig), indicating that pneumococcal esterases can be released outside cells. To determine whether esterase release is due to autolysin activity, esterase activity was tested in clear cell lysates and culture supernatants of an autolysin mutant and wild type pneumococcus. No difference was found in esterase activity in the autolysin mutant (clear cell lysate: 41±3.4 mU and culture supernate: 15±2.7 mU, n = 4) compared to the wild type (clear cell lysate: 42.6±2.3 mU and culture supernate: 17±1.8 mU, n = 4) (p>0.05). This means that the esterase activity in culture supernatant is not due to autolysin activity but due to an unknown export mechanism.

### EstA is involved in pneumococcal colonization of the nasopharynx

**Fig 6:**
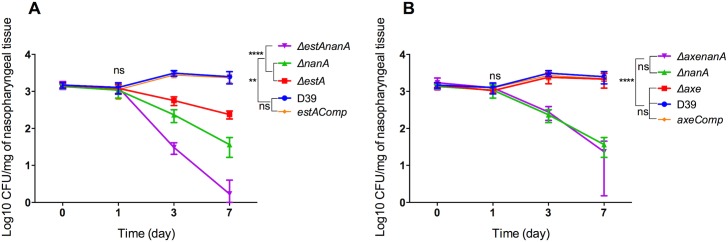
**Assessment of *ΔestA* (A), *Δaxe* (B) and their genetically complemented versions in nasopharyngeal colonization.** Each mouse received approximately 5X10^5^ CFU intranasally in 20 ml PBS. Each time point represents a mean of 5 individual CFU/mg of nasopharyngeal tissue. The vertical bars indicate the standard error of mean. ** p<0.01, **** p<0.0001.

After biochemical assays, and growth studies, next, we evaluated esterases’ role in pneumococcal colonization and virulence, and also assessed whether esterase activity would potentiate neuraminidase A activity *in vivo*. In asymptomatic colonization model, it was found that the number of D39 recovered from nasopharyngeal tissues remained constant over 7 days (3.16 ±0.23 CFU/mg, 3.08 ±0.16 CFU/mg, 3.48 ±0.27 CFU/mg and 3.36 ±0.06 CFU/mg, n = 10 for all at day 0, 1, 3, and 7 days post-infection, respectively) (**Fig 6**). However, at 3 and 7 days post-infection, the numbers of *ΔestA* (Log_10_ 2.73±0.04 and 2.36±0.03 CFU/mg n = 10, respectively) and *ΔnanA* (Log_10_ 2.34±0.05 CFU/mg and 1.5±0.07 CFU/mg n = 10) were significantly lower than D39 (p<0.01). However, the biggest attenuation in colony counts was detected with *ΔestAnanA* (Log_10_ 1.46±0.05 CFU/mg and 0.61 ±0.04 CFU/mg (n = 10) at 3 and 7 days post-infection, respectively). The numbers of *ΔestAnanA* at 3 and 7 days post-infection were significantly lower than those of *ΔestA* and *ΔnanA* (p<0.0001). There was no significant difference in colony counts of genetically complemented strain and the wild type (p>0.05). On the other hand, Axe did not contribute in colonization (**Fig 6)**.

### EstA contributes to pneumococcal virulence

**Fig 7:**
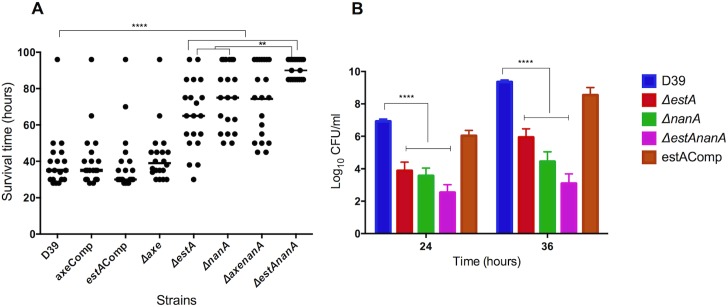
The contribution of esterases in pneumococcal virulence independently and synergistically with neuraminidase A. **A**. Survival of mice infected intranasally with approximately 1X10^6^ CFU *ΔestA* or *Δaxe* isogenic mutants and their genetically complemented derivatives. The horizontal lines refer to the median survival time. Each dot represent the survival time of an individual mouse. **B.** Progression of bacteraemia in mice infected intranasally with pneumococcal strains. Each column represents the mean Log CFU/ml of blood from 10 animals. The vertical bars indicate the standard error of mean. ** p<0.01, ****, p< 0.0001 compared to wild type.

The contribution of EstA and Axe to pneumococcal virulence was determined in a mouse model of pneumococcal pneumonia with bacteraemia that develops after intranasal infection. The results showed that the median survival time of cohorts infected either with *ΔestA* (65 h ±19.6, n = 20) and *ΔnanA* (75 h ±17.4, n = 20) was significantly higher than mice infected with D39 (35 h ±15.2, n = 20) or estAComp (30 h ±16.9, n = 20) (p<0.0001) (**Fig 7A**). Moreover, the median survival time of mice infected with *ΔestAnanA* (90 h ±7.2, n = 20) was significantly higher than either *ΔestA* or *ΔnanA* (p<0.01). On the other hand, there was no difference in survival time of the cohort infected with *Δaxe* (39 h ±15.3, n = 20) and D39 (p>0.05), and no difference was detected in survival time of *ΔnanA* (75 h ±17.4, n = 20) and *ΔaxenanA* (75 h ±20.1, n = 20) infected mice (p>0.05) (**Fig 7A**).

In addition, the progression of bacteraemia was determined after intranasal infection (**Fig 7B**). The numbers of *ΔestA* at 24 and 36 h post-infection (Log_10_ 3.9±0.53 CFU/ml blood and 5.94±0.52 CFU/ml blood, n = 20, respectively) was significantly lower than the numbers of D39 at 24 and 36 h (Log_10_ 6.9±0.13 CFU/ml and 9.4±0.11 CFU/ml, respectively) (p<0.0001). There was no significant difference between estAComp and D39 wild type (p>0.05) indicating that the mutation of *estA* did not cause a polar effect. Similarly, *ΔnanA* (3.56±0.2 CFU/ml and 4.44±0.6 CFU/ml, n = 20, at 24 and 36 h, respectively) and *ΔestAnanA* (2.53±0.48 CFU/ml and 3.1±0.59 CFU/ml, n = 20, at 24 and 36 h, respectively) had lower blood counts than the wild type (p<0.0001) (**Fig 7B**). However, no significant difference in bacterial count between *ΔnanA* and *ΔestAnanA* was seen, suggesting that removal of acetylation may not be critical for pneumococcal growth in blood.

### Expression of *estA* and *axe*

Despite Axe’s ability to potentiate sialic acid release by NanA in cell free system, Axe neither contributed in pneumococcal growth on BSM (**Fig 5B**) nor sialic acid release from BSM (**Fig 5C**). We therefore hypothesised that this could be due to the induction of *estA* expression in *Δaxe*. The increased expression of *estA* in *Δaxe* is plausible because we demonstrated that the expression of both *estA* (4.5±0.66 fold) and *axe* (2.07±0.52 fold) were up-regulated in CDM supplemented with BSM relative to their expression on glucose. *In vivo* expression of *estA* and *axe* was also determined in pneumococci recovered from infected mouse tissues. The result showed that both *estA* and *axe* expression was significantly higher in the mucin rich nasopharynx (12.6±0.7 and 6.5±0.5 folds, respectively) and in the lungs (6.3±0.7 and 1.6±0.5 folds, respectively) relative to their expression in blood (p<0.05). When the expression of *estA* was evaluated in *Δaxe* exposed to BSM, the expression of *estA* increased (7.73±1.21 folds) relative to parental strain exposed to BSM, implying that the loss of *axe* is compensated by *estA* expression. Similarly, *axe* expression in *Δaxe* was also investigated. The analysis revealed that that *axe* expression was not significantly different, 1.3±0.21 fold (n = 3), in *ΔestA* relative to the wild type (p>0.05).

### EstA and NanA induce nasopharyngeal mucin secretion

**Fig 8:**
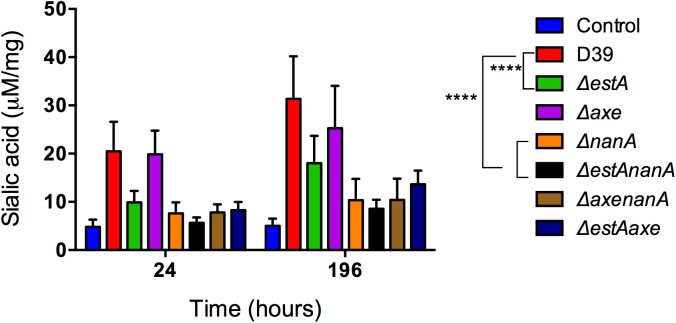
The contribution of EstA and Axe in mucin secretion in nasopharyngeal cavity of mice colonized with different pneumococcal strains. The level of bound sialic acid in nasopharyngeal lavage of mice 7 days post-infection. Mice challenged with PBS served as a negative control. ** p<0.01, *** p< 0.001, ****, p< 0.0001 compared to wild type.

To determine whether EstA and NanA are involved in release of sialic acid *in vivo*, the level of bound sialic acid in nasopharyngeal lavage of mice colonised with different isogenic mutants was assessed (**Fig 8)**. As can be seen from Fig 8, the level of bound sialic acid in mice colonised with wild type D39 (35±3.7 μM/mg, n = 5) was significantly higher than the control mice that had received PBS alone (6.09±4.1 μM/mg, n = 5) (p<0.01), while those colonised with *ΔestA* and *ΔnanA* (23.12±8.1 μM/mg, and 11.65±6.4 μM/mg, n = 5, respectively) had significantly decreased sialic acid concentration compared to the wild type infected cohort (p<0.01 and p<0.0001, for *ΔestA* and *ΔnanA*, respectively). There was no difference in released sialic acid level in the nasopharyngeal washings of mice colonised either with *ΔestAnanA* or *ΔnanA* (p>0.05) (**Fig 8**). In addition, the released sialic acid level in the nasopharyngeal wash of mice colonised with *Δaxe* (25.3±3.6 μM/mg, n = 5) was similar to that of the wild type (**Fig 8**) (p>0.05). As the bound sialic acid level was higher in the nasopharyngeal wash of mice than in the PBS administered cohort, these data suggest that an intact pneumococcal deglycolysation system is required for the sialylated glycoconjugate synthesis.

As colonization experiments indicated that the nasopharyngeal bacterial load differ among the study strains after 3 and 7 days post-infection (**Fig 6**), sialylated glycoconjugate content was also determined at 24 h post-infection to exclude the possibility that observed differences are due to bacterial load. Our results showed that the bacterial load at 24 h post-infection in the nasopharynx was similar among the strains (**Fig 6**), and sialylated glycoconjugate content displayed the same pattern as detected 7 days post-infection (**Fig 8),** ruling out possibility that the release was due to bacterial burden.

## Discussion

O-acetylation hinders the cleavage of sialic acids from glycoconjugates. However, the microbes posses esterase activity to remove acetylation from sialic acids. Our results showed that the pneumococcal esterase activity is coded by multiple genes, indicating that the pneumococcus encounters with acetylated compounds during infection at different tissue sites, and/or during its metabolic processes. EstA is responsible for the main pneumococcal esterase activity, and its expression is induced by BSM, and in respiratory tract. This strongly indicates that EstA is involved in deacetylation of host glycoconjugates. All four pneumococcal esterases studied were found to be specific for short acyl chain esters (Fig 1). This preference for esters of short-chain fatty acids is characteristic of esterases, and it is found to be common among other lactococcal esterases [43].

Among pneumococcal putative esterases, tributyrin esterase (SPD_0534, EstA), part of the core pneumococcal genome [44], has been structurally characterised [45]. Screening of a mutant library indicated that EstA is required for pneumococcal lung infection [46], and it can induce nitric oxide and pro-inflammatory cytokine production in macrophages [47]. However, how EstA contributes to pneumococcal virulence and pathogenesis of disease is not known in detail. By biochemical assays, and *in vitr*o growth studies we demonstrated synergistic action of EstA and NanA on acetylated sialic acid, and their involvement in pneumococcal colonization and virulence. We found that *ΔestAnanA* cleaved less sialic acid from BSM (Fig 5) and had lower colony counts in nasopharynx than *ΔnanA* (Fig 6), reflecting synergistic action of these enzymes. The combined action of these two enzymes results in higher acetate release (Fig 4), and augments the utilization of mucin (Fig 5) probably due to an increase in utilizable sugar availability through the exposure of glycosidic bonds to the activity of pneumococcal glycosidases, which ensures a steady supply of various utilizable sugars such as mannose, N-acetyl glucosamine, and, critically, galactose. Galactose is rich in respiratory mucin, and is one of the crucial sugars for pneumococcal metabolism, colonization and virulence [11, 48]. The pneumococcus requires high concentration of galactose in extracellular milieu due to inefficiency of galactose transporters [11]. Therefore, the effective release of sialic acid ensures continuous provision of galactose for pneumococcus. In addition, the concerted action of EstA and NanA leads to increased cleavage of sialic acid, which is known to be important for pneumococcal biofilm formation, and whose intranasal inoculation significantly increases pneumococcal counts in the nasopharynx and instigates translocation of pneumococci to the lungs [22].

In addition to a nutritional impact of EstA-NanA synergism, the potentiation of neuraminidase efficacy by esterase enhances the microbe’s capacity to colonize and invade respiratory tissues, as we demonstrated in this study (Figs 6 and 7). Neuraminidase activity has been shown to be required for biofilm formation [49], for resistance to opsonophagocytic killing by human neutrophils [50], and for cell attachment by exposing potential ligands for bacterial receptors [51]. The comprehensive impact of neuraminidase is linked to the ubiquitous presence and abundance of sialic acid on the surface of all mammalian cell types. The typical cell is reported to exhibit tens of millions of sialic acid molecules, and it is estimated that the local concentrations on the cell surface glycocalyx can reach up to 100 mM [52]. In this study we selected NanA to test our hypothesis as it has broader substrate specificity than other pneumococcal neuraminidases, and it provides the main pneumococcal neuraminidase activity. It is likely that esterases potentiate the activity of the other neuraminidases coded by *nanB* and *nanC* because esterase will remove acetylation independent of neuraminidase activity.

EstA and NanA activity is important not only for efficient cleavage of acetylated sialic acid but also, as we demonstrated, they are required for sialylated glycoconjugate synthesis in respiratory mucosa (Fig 8). This paradox very likely ensures a steady supply of host glycoconjugates for mucosal commensals such as *S*. *pneumoniae* during nasopharyngeal colonization, while replenishing surfaces of respiratory mucosa with the sialylated glycans to prevent the infiltration of obligate pathogens [8]. The level of sialylated glycan was lower in mice colonised with *ΔnanA* isogenic mutant than those with the wild type, indicating that cleavage of sialic acid is recognised as a signal for respiratory mucin secretion [4, 53].

We demonstrated that Axe could utilise acetylated xylan as a substrate (Fig 3). Therefore, by definition Axe is an acetyl xylan esterase. Acetyl xylan esterases hydrolyse the ester linkages of the acetyl groups in position 2 and/or 3 of the xylose moieties of natural acetylated xylan from hardwood [40]. Xylan is not found in mammalian host. However, structural analogues of acetylated xylan are present in extracellular matrix [54]. Therefore, it is plausible that Axe may have a role in removal of acetylation in mammalian xylan analogues. Contrary to the biochemical assay results, in which Axe could utilize acetylated substrates, no measurable contribution of Axe could be detected in pneumococcal mucin utilization, colonization or virulence in our experimental models. This is very likely to be due to the absence of specific substrates for Axe in BSM, and in mouse tissues.

Subcellular localization assay identified EstA and Axe as intracellular proteins though esterase activity could also be detected in the spent culture supernates as well (S3 Fig). Detection of esterase activity in extracellular milieu is consistent with the reduced growth yield and the released sialic acid concentration by Δ*estA* and Δ*estAnanA* on BSM (Fig 5). Currently, the mechanism of esterase release by pneumococcus is not known. It has been reported that certain pneumococcal intracellular proteins, such as α-enolase and pneumolysin, which do not have a signal peptide, similar to EstA and Axe, can have a cell surface localization [55, 56]. In addition, it is known that the pneumococcal proteins can be released due to autolytic activity [57]. However, there was no difference in esterase activity between autolysin mutant and the wild type, suggesting strongly the presence of an unknown export mechanism.

We investigated the functional importance of putative serine active sites in Axe and EstA. The active site of esterases is usually within the typical catalytic triad of the nucleophile serine, proton carrier histidine and aspartate/glutamate [38]. This typical triad forms a sophisticated pocket responsible for esterases’ catalytic activity [58]. In the majority of esterases, serine and histidine residues are usually present, whereas aspartate/glutamate residues might be absent in some of them [58]. The mutation of these putative sites in EstA and Axe abolished esterase activity, confirming that EstA and Axe activity requires serine active site.

In this study we demonstrated that removal of *O*-acetylation by esterase potentiates pneumococcal neuraminidase activity, and this process contributes to pneumococcal colonization and virulence. Given that there are posttranslational modifications other than acetylation in host glycoconjugates such as sulfations and methylation [32], further work is required to understand their impact on pneumococcal biology. We only investigated esterases role in potentiation of neuraminidase efficacy in this study, hence we cannot rule out their involvement in other processes. In other bacteria, esterases are implicated in other functions such as lipid production, cell attachment and biofilm formation in *P*. *aeruginosa* [59], and conversion of an inactive bacterial toxin into an active form in *Bordetella pertussis* [60]. Therefore, we intend to study esterases’ role in wider aspects of pneumococcal biology.

## Materials and methods

### Bacterial strains and growth conditions

The list of strains used in this study is given in **S1 Table**. *S*. *pneumoniae* D39 was grown either in brain heart infusion broth (BHI), blood agar base (Oxoid, UK) supplemented with 5% (v/v) defibrinated horse blood (Oxoid), or in Chemically Defined Medium (CDM) supplemented with 55 mM glucose or different concentrations of dialysed bovine sub-maxillary mucin (Sigma) [9, 11, 61]. *Escherichia coli* cultures were grown on Luria broth, or Luria agar (Oxoid, UK). Spectinomycin and kanamycin were added at 100- and 250 μg/mL, respectively, for pneumococcal cultures, and for *E*. *coli* ampicillin and kanamycin were used at 100- and 150 μg/mL, respectively.

### Construction of genetically modified strains

*In vitro* mariner mutagenesis was used for the construction of pneumococcal mutants as previously described using the primers and plasmids listed in **S2 Table** and **S3 Table**, respectively [61, 62]. Successful mutation was confirmed by PCR analysis of transformants using transposon-specific primers, MP127 or MP128, with appropriate chromosomal primers, and by sequencing. A representative strain for each mutation was selected, and these were designated as *ΔestA*, *Δaxe*, *ΔSPD1239* and *ΔSPD0932*. To construct the double *ΔestAnanA* and *ΔaxenanA*, the mutated regions from *ΔestA* and *Δaxe* were amplified, and transformed into *ΔnanA*.

pCEP plasmid [63], which is non-replicative in *S*. *pneumoniae*, was used for the introduction of an intact copy of *estA* and *axe* into a transcriptionally silent site in the pneumococcal chromosome as we described previously [61]. The chromosomal integration of intact copies of genes into isogenic mutants was confirmed using malF2 and pCEPR2 primers. One of the transformants for each genetic complementation was designated as estAComp and axeComp, respectively, for further analysis.

The replacement of a predicted catalytic site serine 121 and 181 in EstA and Axe, respectively, to alanine were achieved by splicing overlap extension PCR (SOEing PCR) [64]. For this, two-step PCR was used: the first reaction amplified the left and right-flanking regions of mutagenic site using modified primers (**S2 Table**), and the second reaction joined the left and the right flanks. The joined PCR product then was digested and ligated into pCEP. The recombinant plasmid was sequenced, and transformed into *ΔestA* and *Δaxe*. The resulting strains were designated as estAComp^S121A^ and axeComp^S181A^.

### RNA extraction from bacterial cells, cDNA synthesis, and quantitative RT-PCR

Trizol reagent kit (Invitrogen, UK) was used to isolate the total RNA [65]. SuperScript III reverse transcriptase (Invitrogen, UK) was used to synthesise first strand cDNA using random hexamers at 42°C for 50 min according to the manufacturer’s instructions. cDNA (15 ng) was amplified in a 20 μl reaction volume that contained 1X SensiMix SYBR Master mix (Bioline, UK) and 3 pmol of each primer (**S2 Table**). The transcription level of specific genes was normalized to *gyrB* transcription, which was amplified in parallel with SPD0709F and SP0709R primers. The results were analyzed by the comparative C_T_ method [66].

### Extraction of pneumococcal RNA from infected tissues

Outbred 8-10-week-old female MF1 mice (Charles River, UK) were intranasally infected with 50 μl PBS containing 1x10^6^ D39 pneumococci [15, 67]. When the mice became severely lethargic they were anesthetized and blood was obtained by cardiac puncture [62]. Subsequently, mice were killed by cervical dislocation, and tissues were dissected and homogenized on ice in 10 ml of sterile PBS. Then homogenates were centrifuged to obtain the bacterial pellet as described previously [62]. RNA extraction and purification was done as above.

### Cloning and expression of recombinant proteins

The *estA*, *axe*, *estA*^*S121A*^, and *axe*^*S181A*^ were PCR amplified, and the amplicons were cloned into pLEICS-01 (**S2 Table**). The recombinant plasmids were sequenced to rule out any unintended mutational events. The recombinant plasmids were transformed into *E*. *coli* BL21 (DE3) for expression. The protein expression was done at 25°C, and induced with 0.5 mM IPTG. Recombinant proteins were then purified using Talon Metal Affinity resin (Clontech Inc., UK) as previously described [9]. The definitive identity of the purified recombinant proteins were verified by matrix-assisted laser desorption ionization–time of flight (MALDI-TOF) by PNACL (University of Leicester).

### Enzyme assays

Esterase and neuraminidase activities were assayed using chromogenic substrates. Esterase activity assay was done as previously described using five different p-nitrophenyl esters including p-Nitrophenyl acetate C2 (p-NPA), p-Nitrophenyl butyrate C4 (p-NPB), p- Nitrophenyl hexanoate C6 (p-NPH), p-Nitrophenyl octanoate C8 (p-NPO) and p-Nitrophenyl decanoate C10 (p-NPD) [68]. 2-O-(p-nitrophenyl)-α-d-N-acetylneuraminic acid (p-NP-NANA) was used for neuraminidase activity [15]. The absorbance of released p-nitrophenol was measured photometrically at 405 nm. The protein concentration was measured according to the method described by Bradford, 1976 [69]. One unit of enzyme activity was defined as 1 μmol p-nitrophenol per min per milligram of protein under standard assay conditions.

In addition, esterase activity was assayed using BSM, tributyrin, and acetylated xylan. To determine esterase activity on BSM, 5 mg of BSM was incubated with different concentrations of recombinant esterases in a total reaction volume of 200 μl in PBS, pH 7.5 at 37°C, and the released acetate was assayed enzymatically using a commercial test kit (Megazyme, Ltd., Ireland). A sample of BSM incubated with esterase at 4°C served as a control [36].

To determine the synergistic interaction between esterases and neuraminidase in sialic acid release, 5 mg of BSM (Sigma Aldrich, UK) was dissolved in 200 μ1 of PBS at pH 7.5, and was incubated with 250 U of each recombinant esterase at 37°C for 30 to 120 min. Then, 250 U of recombinant NanA was added into the reaction mixture and further incubated for a pre-determined time. To stop the reaction, the reaction mixture was placed on ice [70]. The released sialic acid was measured using 0.2 M periodate reagent (0.2 M of sodium periodate in 0.1 M H_3_PO_4_, pH 7.4) as previously described [71].

The activity of esterases on tributyrin was determined as described previously [39, 40]. For qualitative determination, 0.5% (v/v) tributyrin was suspended in 50 mM Tris (pH 8.8) and 25 mM CaCl_2_ and then embedded into 2% (w/v) standard agarose. Commercial lipase from *Staphylococcus aureus* was used as a positive control (Sigma Aldrich, UK). The zone of clearance indicated the presence of tributyrin esterase activity. For quantitative assay, 250 U of recombinant esterase was mixed with 0.5% (w/v) tributyrin suspension embedded in 0.8% (w/v) low melting point agarose. The decrease in absorbance at 450 mm was recorded over time. The activity of tributyrin esterase in the sample was quantified using a standard curve generated with known concentrations of *S*. *aureus* lipase.

Acetyl xylan activity was assayed using birchwood xylan as substrate as previously described [72]. Birchwood xylan was prepared by dissolving in dimethyl sulfoxide, and K_3_BO_3_. The mixture was dialysed against running water, and lyophilized. Different concentrations of recombinant esterases was mixed with 150 μl of substrate solution, which was prepared by mixing 30 mM of acetylated xylan with 0.01% (w/v) bromothymol blue as indicator, and 5 mM of sodium phosphate buffer (pH 7.3). The reaction mixture was incubated at 37°C for 20 min, and the absorbance of supernate at 616 nm was recorded. The decrease in absorbance indicated enzyme activity, which was calculated by generating a standard curve using acetic acid. One unit of activity was defined as the formation of 1 μM of acetic acid per minute under the standard reaction medium.

### Subcellular localization of esterases

To determine the cellular localization of esterases, the pneumococcus was grown in CDM supplemented with either 0.071% (w/v) BSM or 55 mM glucose until late exponential phase, when the cell pellets were harvested. The pneumococcal whole cell lysate was separated into cell wall, membrane and cytoplasmic fractions as described before [73]. The recombinant proteins or cellular fractions were separated by SDS PAGE gel, and western blotting was done using polyclonal antibody as previously described [9]. Briefly, ten weeks old female CD1 outbred mice (Charles River, UK) were injected intraperitoneally with a 25 μg of recombinant proteins and 33 μl of Imject Alum adjuvant (Perbio Science, Cramlington, UK) and 67 μl of PBS, while the control group received only adjuvant and PBS. Injections were repeated three times at fortnightly intervals. Two weeks after the last injection, mice were anesthetized with 3% (v/v) isoflurane (Astra Zeneca, Macclesfield, UK) over oxygen (1.5 to 2 liters/min) and blood was collected by cardiac puncture. The blood was left at room temperature for one hour to clot and the serum was recovered by centrifugation, and was kept at -8°C until needed.

### *In vivo* studies

Female 8 to 10 week old MF1 mice weighing approximately 30 to 35 g (Charles River, UK), were anesthetized with 2.5% isoflurane over oxygen (1.5 to 2 litre/min). For carriage model, each mouse was infected intranasally by administering approximately 1x10^5^ CFU of pneumococci in 20 μl PBS. Pneumococcal numbers in nasopharyngeal tissues were determined by plating out the serial dilutions of nasopharyngeal tissue homogenates. Dissection and homogenization of nasopharyngeal tissues were done as described previously [9, 62].

For the pneumonia model, the anesthetized mice were infected intranasally with approximately 1X10^6^ CFU in 50 μl PBS [9, 62]. The inoculum was administered drop-wise. Mice were scored for signs of disease (starry coat, hunched and lethargic) for 7 days [74]. At 24 and 36 hours post-infection, a sample of blood was collected from the tail vein, diluted serially in PBS, and dilutions were plated out to determine bacterial load in the blood. Mice were culled when they manifested lethargic signs, and the time to this point was considered as the survival time.

### Ethics statement

Mouse experiments at the University of Leicester were performed under appropriate project (permit no. 60/4327) and personal (permit no. 80/10279) licenses according to the United Kingdom Home Office guidelines under the Animals Scientific Procedures Act 1986, and the University of Leicester ethics committee approval. The protocol was approved by both the U.K. Home Office and the University of Leicester ethics committee. Where indicated, the procedures were carried out under anesthetic with isoflurone. Animals were housed in individually ventilated cages in a controlled environment, and were frequently monitored after infection to minimize suffering. Every effort was made to minimize suffering and in bacterial infection experiments mice were humanely culled if they became lethargic.

### Statistical analysis

Statistical analysis was determined using Graphpad Prism software 6.0f (Graphpad, California, USA). Data were expressed as means ± standard error of the mean (SEM). One- and two-way analysis of variance (ANOVA) followed by Dunnett's multiple comparison tests were used to compare the groups for enzyme assays and growth analysis. The Mann Whitney test was used for *in vivo* survival experiment whereas one-way ANOVA followed by Tukey's multiple comparisons test was used to compare the groups for bacteremia development and colonization experiment.

## Supporting information

S1 Fig **Amino acid sequence alignment of EstA (A) and Axe (B) with other esterases showing the secondary structure of enzymes.** The blue box represents the conserved consensus sequence and serine active site is located in the centre of the conserved sequence. The purple boxes represent the catalytic residues of Asp and His. The colour range represents the similarity range from high similarity (Red) to low similarity (Mountain blue). The black stars represent the identical residues. Biological sources and accession numbers for the sequences is as follows: SPD_0534: EstA from *S*. *pneumoniae*; llm_1953: tributyrin esterase from *L*. *lactis*; hsa_2098: human esterase D; eco_b2154: S-formylglutathione esterase from *E*. *coli* K12 MG1655; hin_HI0184: S-formylglutathione esterase from *H*. *influenzae* Rd KW20.
(PDF)

S2 Fig **Time dependent EstA (A) and Axe (B) activity using BSM as the substrate.** The assay was set up at pH 7.5 and 250 mU enzyme concentration was used for all reactions. Each column represents the mean of data derived from at least four independent experiments in replicates. The vertical bars show the standard error of mean. The amount of acetate was measured using a commercial kit (Megazyme acetic acid detection kit, Ireland).
(PDF)

S3 Fig Determination of esterases’ subcellular localization in D39 using western blot. The membranes were incubated with polyclonal antibody, which had been raised against Axe and EstA in mice, for 1 hour, and were then incubated with secondary anti-FC antibody for 1 hour. The bands were visualised by NCIP/NBT developing solution. Lane 1: Precision protein ladder; L1, 20 μg of recombinant EstA (≅31kDa) or Axe (≅37kDa); L2, intracellular fractions; L3, membrane fraction; L4, cell wall fraction; L5, supernate hybridised with EstA polyclonal antibody; L6, supernate hybridised with Axe polyclonal antibody.
(PDF)

S1 Table Bacterial strains used in this study. (PDF)

S2 Table Oligonucleotide primers used in this study. *: Spectinomycin mini cassette primer.
**: Italicized nucleotides are incorporated to provide homology to the cloning site in pLEICS-01 plasmid.
***: Italicized nucleotides represent BamHI, Bstz17I and NcoI restriction sites.
****: Bold and italicized nucleotides represent the replacement of thiamine to guanine to change the amino acid serine to alanine.
(PDF)

S3 Table Plasmids used in this study. (PDF)
