# Supplementary material for: Deacetylation of sialic acid by esterases potentiates pneumococcal neuraminidase activity for mucin utilization, colonization and virulence
Source: PLoS Pathog. 2017 Mar 3;13(3):e1006263. doi: 10.1371/journal.ppat.1006263 (PMC5352144; doi:10.1371/journal.ppat.1006263)
Supplement: S1 Fig — Amino acid sequence alignment of EstA (A) and Axe (B) with other esterases showing the secondary structure of enzymes. The blue box represents the conserved consensus sequence and serine active site is located in the centre of the conserved sequence. The purple boxes represent the catalytic residues of Asp and His. The colour range represents the similarity range from high similarity (Red) to low similarity (Mountain blue). The black stars represent the identical residues. Biological sources and accession numbers for the sequences is as follows: SPD_0534: EstA from S. pneumoniae; llm_1953: tributyrin esterase from L. lactis; hsa_2098: human esterase D; eco_b2154: S-formylglutathione esterase from E. coli K12 MG1655; hin_HI0184: S-formylglutathione esterase from H. influenzae Rd KW20. (PDF) [file ppat.1006263.s001.pdf]

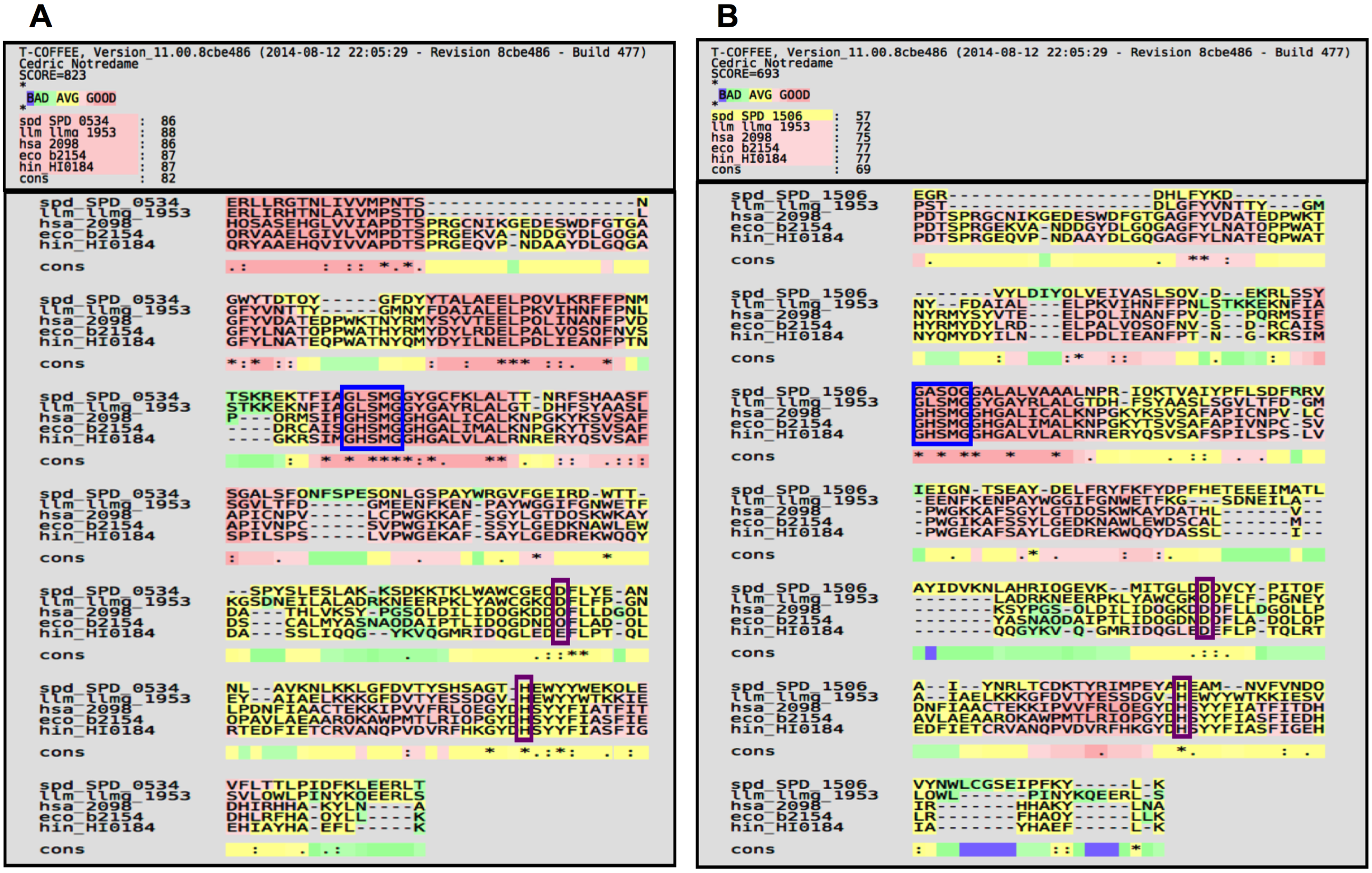
A
T-COFFEE, Version_11.00.8cbe486 (2014-08-12 22:05:29 - Revision 8cbe486 - Build 477)
Cedric Notredame
SCORE=823
BAD AVG GOOD
spd_SPD_0534 : 86
llm_llmg_1953 : 88
hsa_2098 : 86
eco_b2154 : 87
hin_HI0184 : 87
cons : 82
B
T-COFFEE, Version_11.00.8cbe486 (2014-08-12 22:05:29 - Revision 8cbe486 - Build 477)
Cedric Notredame
SCORE=693
BAD AVG GOOD
spd_SPD_1506 : 57
llm_llmg_1953 : 72
hsa_2098 : 75
eco_b2154 : 77
hin_HI0184 : 77
cons : 69
