# Supplementary figures and images for: Deacetylation of sialic acid by esterases potentiates pneumococcal neuraminidase activity for mucin utilization, colonization and virulence
Source: PLoS Pathog. 2017 Mar 3;13(3):e1006263. doi: 10.1371/journal.ppat.1006263 (PMC5352144; doi:10.1371/journal.ppat.1006263)

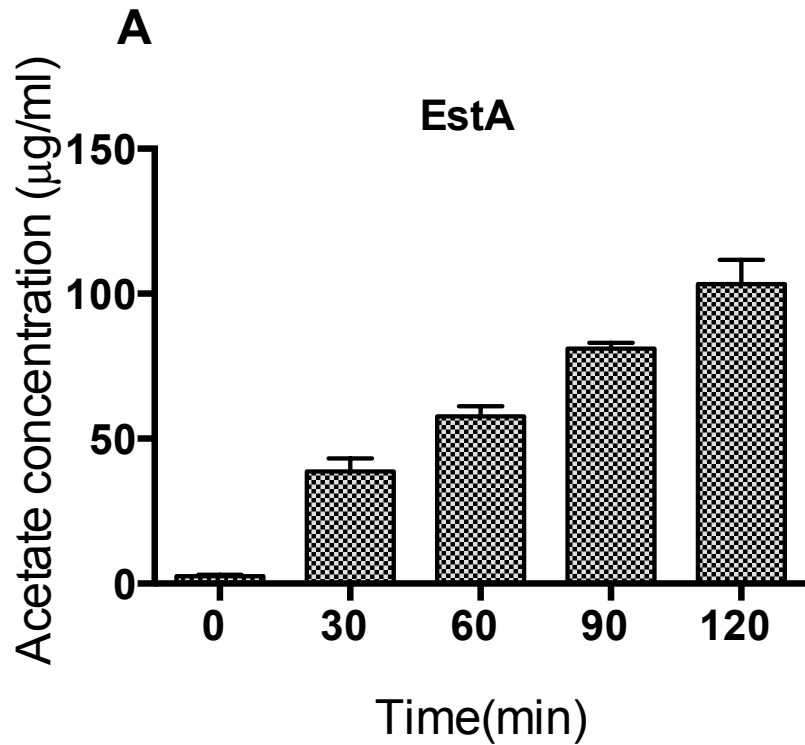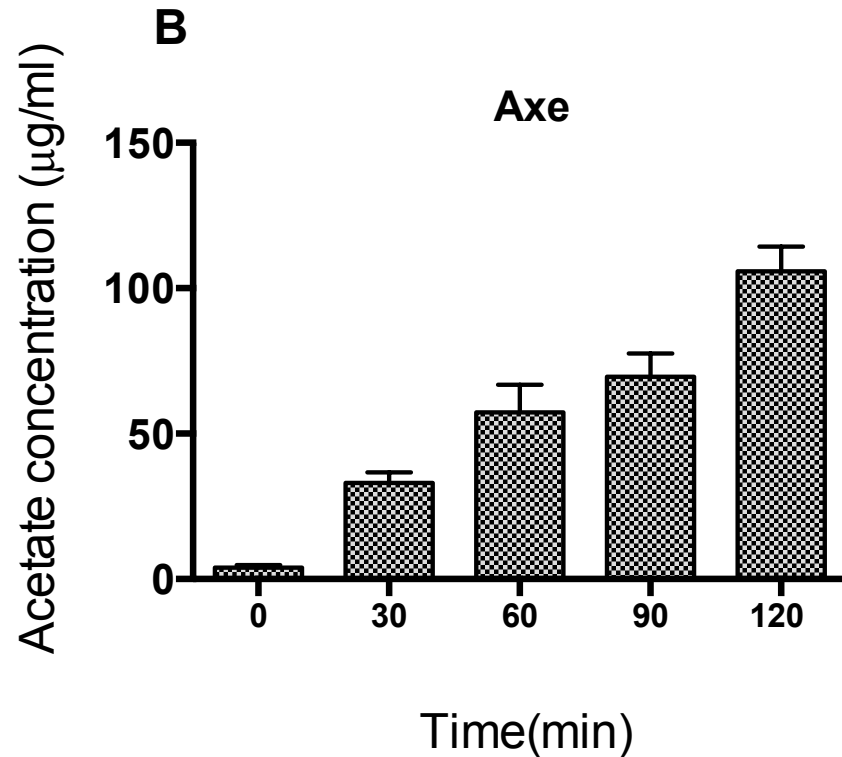

Supplement: S2 Fig — Time dependent EstA (A) and Axe (B) activity using BSM as the substrate. The assay was set up at pH 7.5 and 250 mU enzyme concentration was used for all reactions. Each column represents the mean of data derived from at least four independent experiments in replicates. The vertical bars show the standard error of mean. The amount of acetate was measured using a commercial kit (Megazyme acetic acid detection kit, Ireland). (PDF) [file ppat.1006263.s002.pdf]

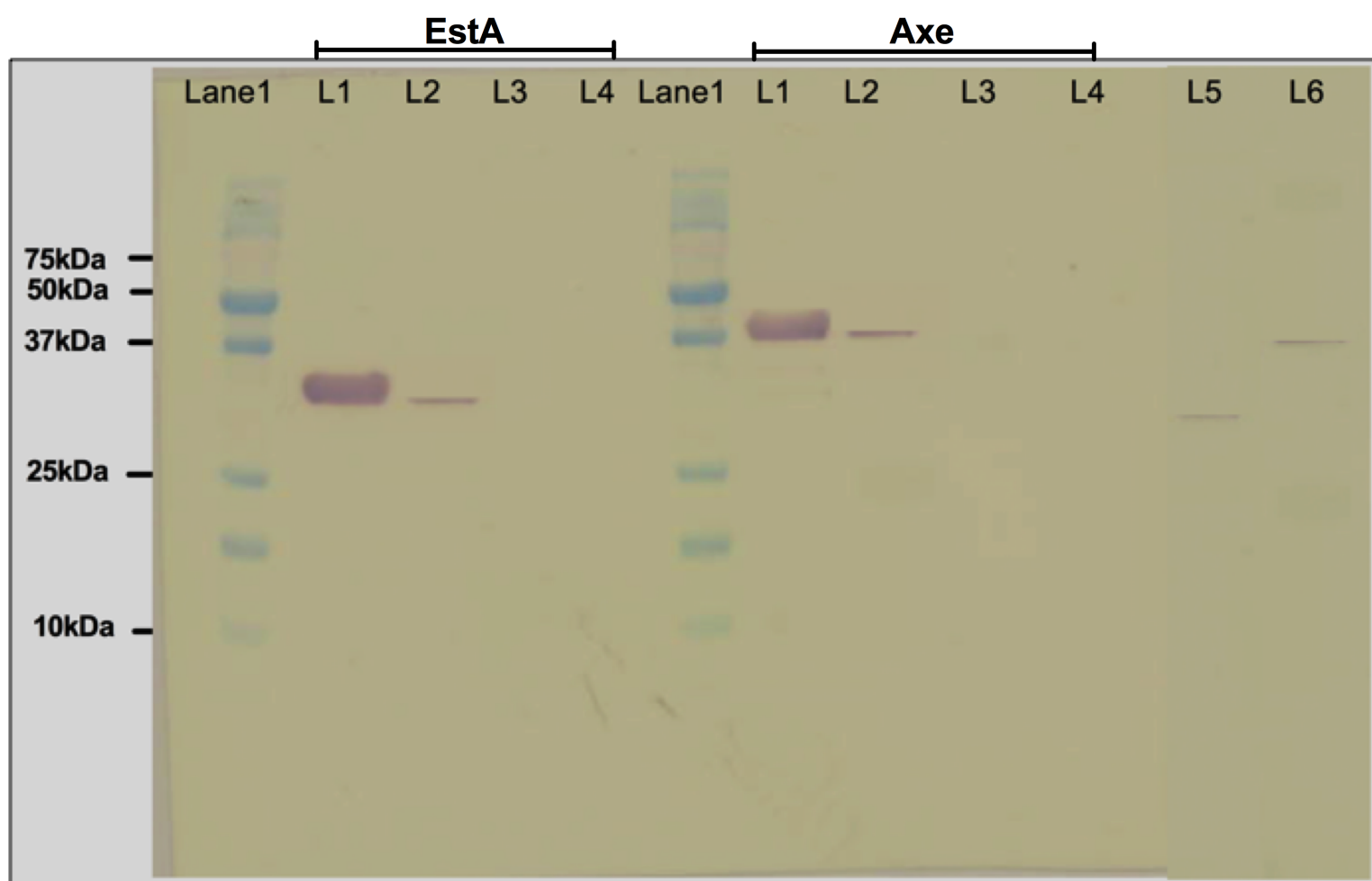

Supplement: S3 Fig — The membranes were incubated with polyclonal antibody, which had been raised against Axe and EstA in mice, for 1 hour, and were then incubated with secondary anti-FC antibody for 1 hour. The bands were visualised by NCIP/NBT developing solution. Lane 1: Precision protein ladder; L1, 20 μg of recombinant EstA (≅31kDa) or Axe (≅37kDa); L2, intracellular fractions; L3, membrane fraction; L4, cell wall fraction; L5, supernate hybridised with EstA polyclonal antibody; L6, supernate hybridised with Axe polyclonal antibody. (PDF) [file ppat.1006263.s003.pdf]
