# Supplementary material for: Deacetylation of sialic acid by esterases potentiates pneumococcal neuraminidase activity for mucin utilization, colonization and virulence
Source: PLoS Pathog. 2017 Mar 3;13(3):e1006263. doi: 10.1371/journal.ppat.1006263 (PMC5352144; doi:10.1371/journal.ppat.1006263)
Supplement: S1 Table — (PDF) [file ppat.1006263.s004.pdf]

Strains	Genotype	Source
E. coli BL21 DE3	*F-ompT hsdsB (rb-mb)gal dcm (DE3)*	Agilent Technology Ltd., UK
E. coli DH5 α	*F⁻, φ 80dlacZ ΔM15, Δ(lacZYA -argF)U169, deoR , recA1 , endA1 , hsdR17 (rK⁻, mK⁺), phoA, supE44 , λ⁻, thi -1, gyrA96 , relA1*	In-Fusion HD Cloning Kit Clontech, USA
S. pneumoniae D39	Virulent type 2 strain	The National Collection of Type Cultures, London, UK Strain NTCC 7466
ΔSPD0534 (*ΔestA*)	Tributyrin esterase, putative	This study
ΔSPD1506 (*Δaxe*)	Acetyl xylan esterase, putative	This study
ΔSPD1239	Acyl-ACP-thioesterase, putative	This study
ΔSPD0932	Phosphoesterase, putative	This study
ΔestAnanA	Tributyrin esterase-neuraminidase A double mutant	This study
ΔaxenanA	Acetyl xylan esterase-neuraminidase A double mutant	This study
ΔestAaxe	Tributyrin esterase-acetyl xylan esterase double mutant	This study
ΔnanA	Neuraminidase A	This study
estAComp	Tributyrin esterase complemented	This study
axeComp	Acetyl xylan esterase complemented	This study
estAComp^{S121A}	Tributyrin esterase modified complemented	This study
axeComp^{S181A}	Acetyl xylan esterase modified complemented	This study
ΔlytA	Autolysin mutant	1

Supporting reference:

1. Balachandran P, Hollingshead SK, Paton JC, Briles DE. The autolytic enzyme LytA of *Streptococcus pneumoniae* is not responsible for releasing pneumolysin. J Bacteriol. 2001;183(10):3108-16.
