# Supplementary material for: Deacetylation of sialic acid by esterases potentiates pneumococcal neuraminidase activity for mucin utilization, colonization and virulence
Source: PLoS Pathog. 2017 Mar 3;13(3):e1006263. doi: 10.1371/journal.ppat.1006263 (PMC5352144; doi:10.1371/journal.ppat.1006263)
Supplement: S2 Table — *: Spectinomycin mini cassette primer. **: Italicized nucleotides are incorporated to provide homology to the cloning site in pLEICS-01 plasmid. ***: Italicized nucleotides represent BamHI, Bstz17I and NcoI restriction sites. ****: Bold and italicized nucleotides represent the replacement of thiamine to guanine to change the amino acid serine to alanine. (PDF) [file ppat.1006263.s005.pdf]

Gene/Symbol	Primer Sequence (5′–3′)		Purpose of use	Source
SPD1506	Forward	TACAGACAAGCCTGACGATAGC	Mutation	This study
	Reverse	TGCATTGATTACATAGATATGC	Mutation	This study
SPD0534	Forward	GTATCAGGGCACGGAAATGT	Mutation	This study
	Reverse	CCACTCTCTTACTGCGAGCAT	Mutation	This study
SP1408	Forward	CGTCACCAAGAGTGTTGGAA	Mutation	This study
	Reverse	CCGGTTTTGAAAGTCGTCAT	Mutation	This study
SP1052	Forward	GATAGCGTTGAATACACCAAAGATT	Mutation	This study
	Reverse	GAGTTCCTCATCCGCTATATATGG	Mutation	This study
MP127 *	Reverse	CCGGGGACTTATCAGCCAACC	Transposon specific	[1]
MP128 *	Forward	TACTAGCGACGCCATCTATGTG	Transposon specific	[1]
SPD1506p/F	Forward	***TACTTCCAATCCATG*AAAAATCCAGCTTTGCTA	Cloning	This study
SPD1506p/R	Reverse	***TATCCACCTTTACTGTCA*TTTTAGATATTTAAAAGGAAT	Cloning	This study
SPD0534p/F	Forward	***TACTTCCAATCCATG*GCAGTGATGAAAATCGAGTA	Cloning	This study
SPD0534p/R	Reverse	***TATCCACCTTTACTGTCA*AGTCAGTCTCTCTTCTAATT	Cloning	This study
SPD0534C/F	Forward	***CATG*CCATGG*TCGTTGAAATAAACAGAGAG	Genetic complementation	This study

SPD0534C/R	Reverse	***ACG*GGATCC*CTAAGTCAGTCTCTCTTCTAAT	Genetic complementation	This study
SPD1506C/F	Forward	***CATG*GTATAC*GCTTTCTCTAAGGAAAACTT	Genetic complementation	This study
SPD1506C/R	Reverse	***ACG*GGATCC*TTATTTTAGATATTTAAAAG	Genetic complementation	This study
malF2	Forward	GTCAACTGTAGTGGGTTGAAGTCAGC	pCEP sepcific	[2]
pCEPR2	Reverse	CCTTTTCCCGTTCCACATCATAGGTG	pCEP sepcific	[2]
SPD0534S-A/F	Forward	****CGCTGGTCTT***G***CTATGGGAGGCT	Sequencing	This study
SPD0534S-A/R	Reverse	****AGCCTCCCATAG***C***AAGACCAGCG	Sequencing	This study
SPD1506S-A/F	Forward	****CTATGGTGCC***G***CACAAGGAGGGG	Sequencing	This study
SPD1506S-A/R	Reverse	****CCCCTCCTTGTG***C***GGCACCATAG	Sequencing	This study
SPD1239-RT/F	Forward	GAAACGGAAGCCTTGAG CTA	Gene expression	This study
SPD1239-RT/R	Reverse	CGGTCCATGAGAACAAA GGT	Gene expression	This study

EstA-RT/F	Forward	GGCTTAAGCGGACCAAT GTA	Gene expression	This study
EstA-RT/R	Reverse	CAGAACCTGTGGCAATTC CT	Gene expression	This study
SPD0932-RT/F	Forward	AGTGCAGCAGATAAATTG CAT	Gene expression	This study
SPD0932-RT/R	Reverse	AGTGCAGCAGATAAATTG CAT	Gene expression	This study
Axe-RT/F	Forward	GGGATGGGGAAGTGAAA AAT	Gene expression	This study
Axe-RT/R	Reverse	ATGCGTGCATAGACCTTT CC	Gene expression	This study
NanA-RT/F	Forward	GTGAAAATGGGATGGTCCAC	Gene expression	This study
NanA-RT/R	Reverse	CACTTTCGCTTCGGTAGG AG	Gene expression	This study
gyrB-F	Forward	TGATGACCGATGCCGATG	Gene expression	This study
gyrB-R	Reverse	TTGGGCAATATAAACATAACCAGC	Gene expression	This study

Supporting references:

1. Terra VS, Homer KA, Rao SG, Andrew PW, Yesilkaya H. Characterization of novel beta-galactosidase activity that contributes to glycoprotein degradation and virulence in *Streptococcus pneumoniae*. Infect Immun. 2010;78(1):348-57.
2. Guiral S, Henard V, Laaberki MH, Granadel C, Prudhomme M, Martin B, et al. Construction and evaluation of a chromosomal expression platform (CEP) for ectopic, maltose-driven gene expression in *Streptococcus pneumoniae*. Microbiology. 2006;152(Pt 2):343-9.
