# Supplementary material for: Deacetylation of sialic acid by esterases potentiates pneumococcal neuraminidase activity for mucin utilization, colonization and virulence
Source: PLoS Pathog. 2017 Mar 3;13(3):e1006263. doi: 10.1371/journal.ppat.1006263 (PMC5352144; doi:10.1371/journal.ppat.1006263)
Supplement: S3 Table — (PDF) [file ppat.1006263.s006.pdf]

Plasmids/phenotype	Aim of use	Source
pR412/spectinomycin resistance	Mariner mutagenesis	[1]
pCEP/ Kanamycin resistance	SOEing mutagenesis and complementation	[2]
pLEICS-01/ Ampicillin resistance	Proteins expression	PROTEX, University of Leicester

Supporting references:

1. Martin B, Prudhomme M, Alloing G, Granadel C, Claverys JP. Cross-regulation of competence pheromone production and export in the early control of transformation in *Streptococcus pneumoniae*. Mol Microbiol. 2000;38(4):867-78.

2. Guiral S, Henard V, Laaberki MH, Granadel C, Prudhomme M, Martin B, et al. Construction and evaluation of a chromosomal expression platform (CEP) for ectopic, maltose-driven gene expression in *Streptococcus pneumoniae*. Microbiology. 2006;152(Pt 2):343-9.
